# Development and analytical validation of a next-generation sequencing based microsatellite instability (MSI) assay

**DOI:** 10.18632/oncotarget.27142

**Published:** 2019-08-27

**Authors:** Sarabjot Pabla, Jonathan Andreas, Felicia L. Lenzo, Blake Burgher, Jacob Hagen, Vincent Giamo, Mary K. Nesline, Yirong Wang, Mark Gardner, Jeffrey M. Conroy, Antonios Papanicolau-Sengos, Carl Morrison, Sean T. Glenn

**Affiliations:** ^1^OmniSeq Inc., Buffalo, NY 14203, USA; ^2^Center for Personalized Medicine, Roswell Park Comprehensive Cancer Center, Buffalo, NY 14263, USA; ^3^Department of Molecular and Cellular Biology, Roswell Park Comprehensive Cancer Center, Buffalo, NY 14263, USA; ^*^These authors contributed equally to this work

**Keywords:** next-generation sequencing, NGS, microsatellite instability, MSI

## Abstract

**Background:**

We have developed and analytically validated a next-generation sequencing (NGS) assay to classify microsatellite instability (MSI) in formalin-fixed paraffin-embedded (FFPE) tumor specimens.

**Methodology:**

The assay relies on DNA-seq evaluation of insertion/deletion (indel) variability at 29 highly informative genomic loci to estimate MSI status without the requirement for matched-normal tissue. The assay has a clinically relevant five-day turnaround time and can be conducted on as little as 20 ng genomic DNA with a batch size of up to forty samples in a single run.

**Results:**

The MSI detection method was developed on a training set (*n =* 94) consisting of 22 MSI-H, 24 MSS, and 47 matched normal samples and tested on an independent test set of 24 MSI-H and 24 MSS specimens. Assay performance with respect to accuracy, reproducibility, precision as well as control sample performance was estimated across a wide range of FFPE samples of multiple histologies to address pre-analytical variability (percent tumor nuclei), and analytical variability (batch size, run, day, operator). Analytical precision studies demonstrated that the assay is highly reproducible and accurate as compared to established gold standard PCR methodology and has been validated through NYS CLEP.

**Significance:**

This assay provides clinicians with robust and reproducible NGS-based MSI testing without the need of matched normal tissue to inform clinical decision making for patients with solid tumors.

## INTRODUCTION

Microsatellite instability (MSI) is a well-described phenomenon characterized by the altered length of short repetitive regions of DNA referred to as microsatellites. The usual setting of microsatellite instability is deactivation of a mismatch repair system protein [1]. Typically, to determine MSI five microsatellites are tested, usually two mononucleotide repeat markers (BAT-25, BAT-26) and three dinucleotide repeat markers (D2S123, D5S346, and D17S250). After amplification, fragment analysis chromatograms for each microsatellite are manually reviewed to assess differences between tumor and normal samples from the same patient in order to identify length differences, or so-called instability, in each microsatellite. A case with instability in at least 2 of 5 microsatellites is defined as microsatellite unstable “high” or “MSI-H” [2].

Approximately 20% of colorectal adenocarcinomas (CRA) and 30% of endometrial endometrioid adenocarcinomas (EEM) are microsatellite unstable, with the majority being sporadic in nature. A minority of cases are associated with Lynch syndrome, which is characterized by early-onset CRA with right-sided predominance that can be synchronous or metachronous, and an increased incidence of extracolonic neoplasms, including EEM. Although CRA and EEM account for the majority of microsatellite unstable tumors, microsatellite instability has a low but substantial incidence in various other tumors [3–7].

Beyond its function as a screening test to identify patients with Lynch syndrome, MSI status is a critical biomarker of response to checkpoint inhibitors [8, 9]. MSI testing has been FDA-approved as a companion diagnostic for nivolumab monotherapy and nivolumab/ipilimumab combination therapy in CRA. In addition, MSI testing is an FDA-approved companion diagnostic for pembrolizumab immunotherapy across all solid tumors [10–12].

An important weakness of the traditional fragment analysis approach for the detection of MSI status is its inherent need to test a tumor sample in parallel with matched normal tissue, which is often not clinically available. Consequently, the requirement of normal DNA can limit the number of patients that can have testing performed due to the difficulty to obtain normal tissue leading to suboptimal turnaround time or an inability to complete microsatellite testing. Although there are additional published studies that use NGS testing to evaluate MSI status [13–15] and those that use conventional fragment analysis without matched normal, [16, 17] we have developed the first agile NGS platform that is Clinical Laboratory Improvement Amendments (CLIA) certified and New York State Clinical Laboratory Evaluation Program (NYS-CLEP) approved for clinical MSI testing in patients using next-generation sequencing (NGS) that can be utilized across all solid tumors without the need of matched normal tissue [18].

## RESULTS

### Assay development

A broad pool of microsatellite instability markers previously identified by NGS in > 300 solid tumors of various histologies was considered as potential targets for inclusion in the MSI NGS assay [14]. To confirm use of these microsatellite repeat regions as viable MSI NGS markers, we examined NGS data from 28 cases assayed by WES representing 7 MSI-H, 7 microsatellite instability low (MSI-L), and 14 microsatellite stable (MSS). Variant calling of the WES BAM files resulted in 233,269 indel loci detected across the 28 samples. For each indel with a specific reference allele, alternate allele and homopolymer repeat number, a fisher’s exact test was performed to test for difference in proportion for MSI positive (MSI-H) cases and MSI negative cases (MSI-L and MSS). Stringent filtering was further applied, where, unique homopolymer indels (alt allele length range 5–7 bp) with very highly significant fisher’s exact test *P* value < 0.0001 that were present in ≥ 80% (at least 6 out of 7) MSI-H cases but not in MSS cases were identified. The resultant set of 40 loci representing 21 chromosomes were included in the MSI NGS panel design (Supplementary Table 1).

### Development of MSI NGS caller

**Figure 1:**
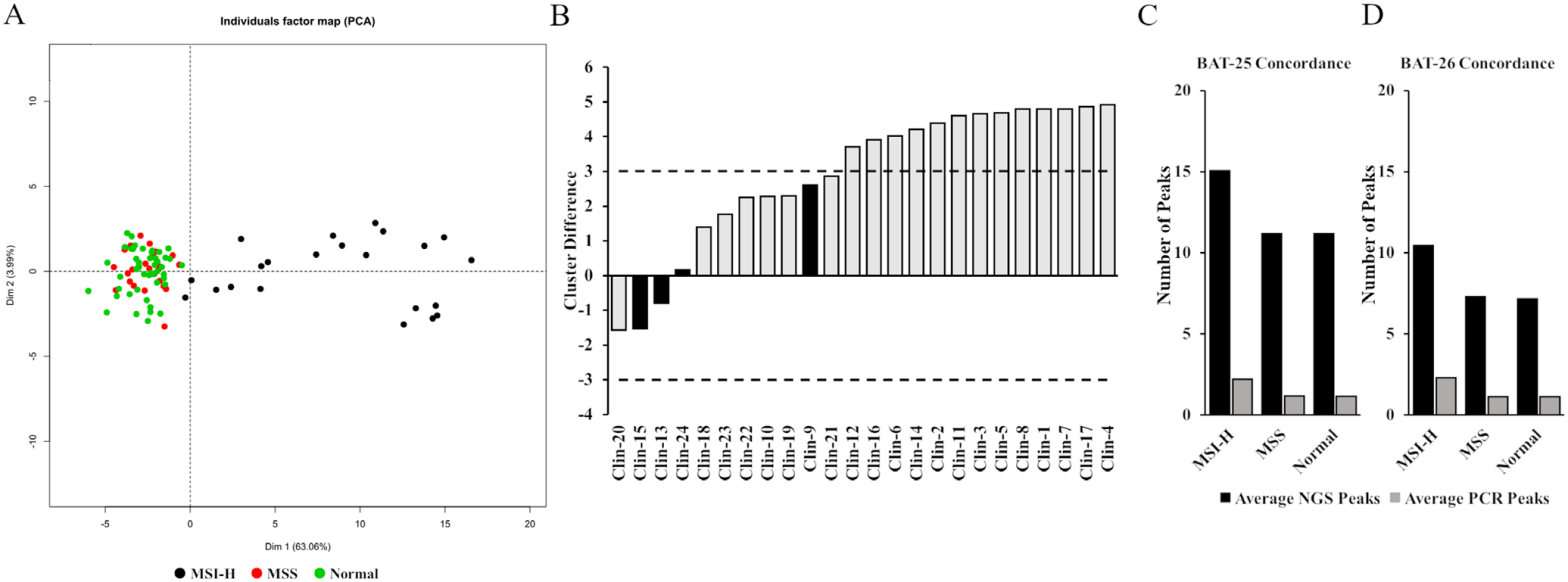
Development and assessment of the MSI NGS caller. (**A**) Principle component analysis was used to visualize separation of 94 MSS, MSI-H and normal training cases that were run by MSI-PCR and MSI NGS. (**B**) 11 out of 24 samples with MSI-PCR data fell between a ±3 centroid distance between cluster 1 and 3. Four had discordant MSI-PCR and MSI NGS calls (black). The inconclusive range was set at > –3 to < 3 (dashed lines). (**C** and **D**) The average number of peaks identified in MSI-H, MSS and normal samples by MSI NGS and MSI-PCR for the two shared Bethesda markers.

MSI NGS Caller was developed using a training dataset of 94 FFPE samples which included 22 MSI-H and 25 MSS samples, and matched normal, as previously determined by gold standard MSI-PCR [19, 20]. Indels were called from mapped BAM files using TNScope v201711.02 (Sentieon Inc., Mountain View, CA, USA). For each homopolymer locus, the number of peaks (count of various indel lengths at same locus) and average indel length (mean of indel lengths at each locus) was calculated (Supplementary Table 2). Out of 522 loci, 29 loci consistently generated peaks data for > 80% of the cases in the training set (Supplementary Table 3), including BAT-25 and BAT-26 PCR markers. As a result, these 29 highly prevalent loci were chosen for further validation and MSI analyses, wherein, for each locus, the number of peaks and average indel lengths of the 94 training cases were used as input for principal component analyses (Supplementary Table 2, subset). PCA was used to visualize a clear separation of MSI-H cases from MSS as well as matched normal (Figure 1A). Next, unsupervised clustering using “k-means” clustering algorithm was performed with k = 3 (3 centroids). “k” was set at 3 to capture a wide spread of the MSI-H group in two separate clusters with only one cluster expected for MSS and matched normal cases (Supplementary Table 4). K-means algorithm works iteratively to cluster each data point to one of K groups based on the 58 features similarity. Each centroid of a cluster is a collection of feature values which define the resulting cluster. Resulting cluster 1 and cluster 2 were assigned as “MSI-H” and cluster 3 was assigned as “MSS”. For classifying test data set as well as other study samples, this training k-means cluster model was used, wherein, 58 features of the test samples were assigned class label of the closest centroid based on its Euclidean distance from all three centroids.

### Defining an inconclusive range

As with all assays that require a set threshold to determine outcome of reporting, true clinical samples that reside close to this decision boundary have the ability to be inaccurately called. Specifically, to the development of this MSI NGS assay, an inconclusive range is necessary to protect against both false positive reporting of MSS cases as MSI-H (subjecting patients to unnecessary treatment) and false negative reporting (lending to missed therapy). As the centroid distances for cluster 1 (MSI-H) and cluster 3 (MSS) get closer together, the ability of the assay to resolve MSS from MSI-H decreases. Comparing MSI-PCR and MSI NGS calls and reviewing the proximity of the cluster 1 (MSI-H) and cluster 3 (MSS) centroid distances to each other allowed for the identification of discordant reporting compared to the MSI-PCR gold standard. Of the 24 samples with MSI-PCR data, 11 cases resided between a ±3 centroid distance between cluster 1 and 3. Of these 11 samples, 4 had discordant calls when comparing MSI-PCR to MSI NGS accounting for an approximate discordance of 37% within the ±3 centroid range (Figure 1B). A critical observation when reviewing the disparate samples is the fact that two of the MSI NGS samples were reported as MSI-H but were reported by MSI-PCR as MSS (false positive reporting), which has critical implications in treatment of patients and further emphasizes the need for an inconclusive range in testing. Alternatively 13 samples that had a centroid cluster difference > 3 between centroid 1 and 3 had 100% concordance. Therefore, the boundary for the inconclusive range of the MSI NGS assay was set at > –3.0 to < 3.0.

### Assessment of MSI NGS caller

To evaluate the performance of the MSI NGS caller, it was first applied to the training set of 94 samples (Supplementary Table 5). Within this cohort eight samples fell into the pre-defined inconclusive category accounting for ~8.5% of all samples tested (Supplementary Table 5, highlighted samples). Of these eight inconclusive samples, two (25%) were MSI-H by MSI-PCR (Supplementary Table 5, bolded samples). For the remaining 86 samples, the MSI caller performed with an accuracy of 100% on this cohort with no false reporting (Table 1). Performance was then assessed using a separate validation set of 47 cases with 23 MSI-H and 24 MSS previously tested using the same clinically approved MSI PCR assay (Supplementary Table 6). Within the validation set six samples fell into the inconclusive category (~12.8%) (Supplementary Table 6, highlighted samples). The MSI caller performed with an accuracy of 100% on this separate validation cohort with no false positive or false negatives reported (Table 1).

**Table 1:** Performance of MSI method on training and validation cohorts

**Training Cohort (94 cases)**	**TP**	**FP**	**TN**	**FN**	**Inconclusive**	**Total**	**Sensitivity**	**Specificity**	**PPV**	**NPV**	**Accuracy**
	20	0	66	0	8	94	100%	100%	100%	100%	100%
**Validation Cohort (47 cases)**	**TP**	**FP**	**TN**	**FN**	**Inconclusive**	**Total**	**Sensitivity**	**Specificity**	**PPV**	**NPV**	**Accuracy**
	17	0	24	0	6	47	100%	100%	100%	100%	100%

To further corroborate concordance with the MSI-PCR assay, we used the two shared “Bethesda” markers^10^ BAT-25 and BAT-26 included in the NGS assay for samples with matched normal available. The number of peaks by NGS and PCR were calculated for tumor and matched normal cases determined as unstable for BAT-25 by PCR assay (Supplementary Table 7). This analysis showed 17 out of 18 (94%) BAT-25 unstable cases with difference in number of peaks (or unique indels present at each loci) for both PCR and NGS assay, demonstrating very high concordance between the two assays. Similarly, 19 out of 20 (95%) BAT-26 unstable cases showed difference in number of peaks for both PCR and NGS assay (Supplementary Table 8). The numerical shift in difference in number of peaks by both methods can be attributed to greater sensitivity of the NGS assay coupled with a calling algorithm designed to accurately call indels in repeat regions. The average number of peaks by NGS and PCR for both BAT-25 and BAT-26 for MSI-H, MSS and Normal groups supports a potential increased sensitivity offered by the NGS assay (Figure 1C and 1D).

### Analytical validation

As part of the analytical validation the robustness, or measure of the MSI NGS assay’s ability to remain unaffected by small variations in procedural parameters, was evaluated. To determine assay robustness, the MSI NGS limit of detection (LOD) was evaluated using serial dilutions of MSI-H Control cell line DNA with MSS Control cell line DNA as well varying levels of tumor DNA with matched normal DNA to assess proportion of malignant tissue required for testing. Additionally, studies to evaluate potential interferents, including variable nucleic acid input and batch size, on microsatellite instability detection were performed (Table 2). These studies included multiple solid tumor specimens representing both microsatellite stable (MSS) and MSI-H phenotypes in addition to a no template control (NTC), MSI-H control (MSI-CTL), and MSS control (MSS-CTL) sample.

**Table 2:** Summary of assay robustness studies

Study section	Design summary	Demonstration
Serial Dilutions (LOD)	MSI-CTRL DNA mixed with MSS-CTRL DNA	Evaluate effect of synthetic percent tumor nuclei (range: 100% MSI-H to 100% MSS) on MSI calling, determine LOD
Tumor Content (LOD)	4 MSI-H Tumor DNA samples mixed with matched Normal DNA	Evaluate effect of percent tumor nuclei (100, 75, 50, 40, 30, 20, 10%) on MSI calling, determine LOD
Variability in DNA input quantity	5 MSI-H samples serially diluted for DNA input	Evaluate effect of DNA input (50, 20, 10, 5 ng) on MSI calling
Batch size	40 libraries (20 MSI-H and 20 MSS) tested as 5, 10, 20, 40 batch sizes	Evaluate effect of batch size on MSI calling as a result of number of samples sequenced per run

#### Level of detection (LOD)

**Figure 2:**
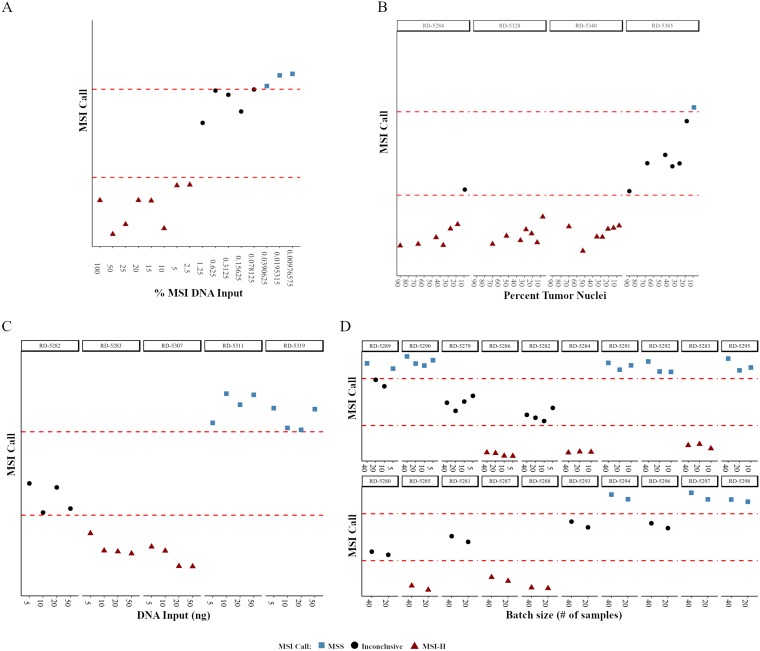
The effects of small variations in procedural parameters on the robustness of MSI NGS calls. NGS calls are plotted as a relative distance to the boundaries of the inconclusive cluster difference (dashed red lines). (**A**) MSI NGS call at decreasing amounts of MSI-H positive DNA mixed with normal DNA. (**B–D**) MSI NGS calls across decreasing tumor content (B), varying amounts of DNA input (C) and sequencing batch sizes (D). RD-# are unique, deidentified clinical patient samples used for testing.

To begin to determine the level of detection of the MSI NGS assay serial dilutions of MSI-H positive DNA extracted from commercially available cell lines were diluted with normal; i.e., non-MSI DNA. The MSI-H DNA contribution ranged from 0.0098% to 100% with the MSI NGS assay having the ability to identify and call MSI-H status down to 2.5% input levels (Figure 2A). As expected, the shift from MSI-H to MSS calling was in alignment of the centroid distance (difference between cluster 1 and 3) falling below the 3.0 threshold that defines our inconclusive range further confirming that the sensitivity of the assay lacks resolution in this inconclusive range.

As the clinical LOD for MSI-H is based on the tumor content of the sample we further determined the LOD of the MSI NGS assay in regards to tumor content using four samples selected from the 50 MSI-H gold-standard samples with sections containing areas of both tumor (70–90% tumor nuclei content) and adjacent normal tissue. To perform this evaluation, MSI-H tumor samples with abundant adjacent normal for which both elements were independently processed for DNA isolation. Tumor content was defined through pathological assessment of the tumor fraction, representing a range of neoplastic cells. The non-tumor DNA was mixed with the tumor DNA to represent a function of decreasing tumor content for two MSI-H samples. A series of seven different percent tumor nuclei dilutions were carried out on the four samples (Supplementary Table 9). For each sample, at the varying percent tumor nuclei amounts, QC data was collected and the number of peaks and mean indel length per loci were calculated to determine the MSI status (Supplementary Table 9). Correlation values were high between the sample specific indel peak number (mean r = 0.932) and mean indel lengths (mean r = 0.924) for the different percent tumor nuclei values, with the MSI NGS calls for three of the four samples within the dilution series maintaining 100% concordance down to 7–9% tumor content (Figure 2B). As the RD-5365 18% dilution sample failed QC (Supplementary Table 9, highlighted) and was used for subsequent serial dilution to 9% these data points were excluded from interpretation. The three samples that passed QC maintained accurate calling of MSI-H status down to 7–9%, therefore the LOD for the MSI NGS assay for clinical testing has been set at 10% tumor nuclei.

#### Variability in DNA input

Inconsistency in the DNA input amounts can be expected in normal practice due to potential variability at the lab bench. To evaluate the potential impact of such variability, the effect of DNA input at 50, 10, and 5 ng compared to standard input of 20ng was performed on five samples (three MSI-H and two MSS; Supplementary Table 10). Correlation values were calculated for both number of peaks and mean indel length when comparing the 20 ng input for each sample (Supplementary Table 10). When comparing the MSI NGS call for each sample across the four DNA input amounts there was 100% concordance (Figure 2C). Although there was high concordance across MSI NGS calls for all DNA input amounts, the standard input of the assay is 20 ng.

#### Batch size

In routine clinical testing, variability in batch size can be expected. To demonstrate that the MSI NGS assay results are unaffected by the number of samples included in a batch (sequencing run), the concordance of MSI NGS calling with varying numbers of samples included per run (5, 10, 20 and 40 samples) was characterized. Forty samples representing both MSS and MSI-H status were run on a single flow cell, followed by a subset of 20, 10, and 5 samples, with each subset run on a single flow cell. As the twenty sample run size is considered optimal for the workflow in the laboratory, correlation values were calculated and shown to be very high for both number of peaks and mean indel length when comparing the 20 sample batch size to the 5, 10, and 40 sample batch sizes for each sample (Supplementary Table 11). When comparing MSI calling for each sample run within the different batch sizes, there was a very high concordance (97.5%) across all runs (Figure 2D). Sample RD-5289 was the only sample to show disparity across final MSI NGS calling due to the 10 sample and 20 sample batch sizes being assigned inconclusive status due to centroid distance (Figure 2D, Supplementary Table 11), whereby in both cases the proximity of this value was very close to the threshold. The consistent MSI NGS results demonstrates the ability of the assay to be performed in batch sizes limited to a maximum of 40 per run, but allow for as few as 5 samples per run depending on volume in the laboratory.

### MSI NGS precision (reproducibility studies)

Precision of the MSI NGS assay was determined through a series of reproducibility experiments to ensure the test’s ability to make concordant calling across variables typically found in routine clinical testing (intra-run, inter-run, inter-operator, inter-day, and inter-barcode variance). Furthermore, this study defined the utility and thresholds associated with a common set of control samples (NTC, MSI-CTL, and MSS-CTL) which are to be included in every run (Table 3). To measure intra-assay precision, six DNA samples were run in triplicate within a run using a single operator. Inter-assay precision was determined by running the same DNA samples without replication on a different day by the single operator, with different barcodes on a different day for the same operator, and on a different day with different barcodes for a different operator (Table 3). This study was designed to independently measure the precision of the analytic steps (reproducibility from DNA) with the result (MSS, MSI-H, or Inconclusive) derived from the MSI NGS caller.

**Table 3:** Summary of reproducibility validation studies

Reproducibility studies		
**Study section**	**Design summary**	**Demonstration**
intra-run variance	6 libraries (3 MSI-H + 3 MSS) + controls tested in triplicate in a single run	Evaluate change in MSI calling as a result of sequencing and bioinformatics pipeline variability in a single run
inter-run variance tech 1, day 1	20 libraries (10 MSI-H + 10 MSS) + controls tested 1x across multiple runs	Evaluate change in MSI calling as a result of sequencing and bioinformatics pipeline variability across multiple runs
inter-run variance tech 2, day 2	20 libraries (10 MSI-H + 10 MSS) + controls tested 1x across two different operators	Evaluate change in MSI calling as a result of sequencing and bioinformatics pipeline variability across 2 operators
inter-run variance tech 1, day 3	20 libraries (10 MSI-H + 10 MSS) + controls tested 1x across two days	Evaluate change in MSI calling as a result of sequencing and bioinformatics pipeline variability across 2 days
between barcodes	20 libraries (10 MSI-H + 10 MSS) + controls tested with multiple barcodes	Evaluate change in MSI calling as a result of sequencing and bioinformatics pipeline variability across multiple barcodes
MSI Run controls	NTC (library QC only), MSI-H and MSS run controls used as template for multiple reproducibility evaluations	Evaluate change in MSI calling as a result of sequencing and bioinformatics pipeline variability across multiple replicates

#### Intra-run variance

The intra-run variance reproducibility study was completed by operator 1 on day 1 using DNA from 6 samples (3 MSI-H and 3 MSS) processed in triplicate with rotating barcodes (Table 3). For all samples run as part of the reproducibility studies QC metrics were calculated (Supplementary Table 12).

**Figure 3:**
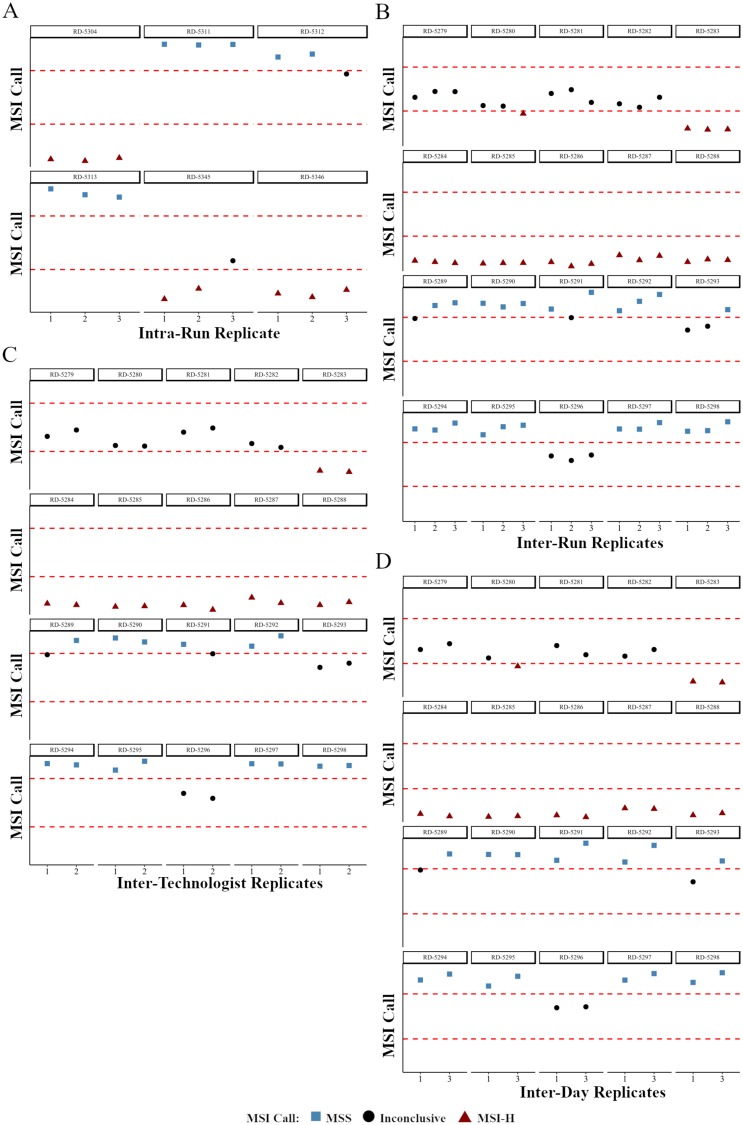
The effects of variables found in routine clinical testing on the precision of MSI NGS calls. NGS calls are plotted as a relative distance to the boundaries of the inconclusive cluster difference (dashed red lines). High concordance of MSI NGS calls is observed with sample replicates from intra-run (**A**), inter-run and barcode (**B**), inter-technologist (**C**), and inter-day (**D**) reproducibility studies. RD-# are unique, deidentified clinical patient samples used for testing.

For each sample of the intra-run reproducibility study the number of peaks and mean indel length per loci were calculated to determine the MSI status and correlations for these parameters were calculated across the replicate sets (Supplementary Table 13). Although for three individual replicates within RD-5312 and RD-5345 an inconclusive call was made due to centroid cluster distance residing very close to the cut-off (Figure 3A, Supplementary Table 13), the intra-run reproducibility study demonstrated 100% concordance when comparing the MSI NGS calls when reviewing the cluster designations between the three replicates for the six samples evaluated (Supplementary Table 13). As the inconclusive range is set to protect against miscalling, reproducibility was defined as the assay’s ability to not call a MSI-H case MSS and vice versa, whereas inconclusive calling for a sample is deemed acceptable especially in cases where the centroid distance resides closely to the decision boundary.

#### Inter-run and barcode variance

Between run reproducibility was performed on 20 samples (10 MSI-H and 10 MSS) across 2 operators, 3 different days, on 3 different runs with each replicate using a different barcode. Briefly, on day 2, operator 1 completed libraries for 20 samples sequenced without replication. On day 3, operator 2 completed a second library for the 20 samples that was again sequenced without replication. On day 3, operator 1 completed a third library that was sequenced without replication for a total of three inter-run DNA replicates for the 20 samples.

For each sample of the inter-run and barcode reproducibility study the number of peaks and mean indel length per loci were calculated to determine the MSI status including correlation across the replicate sets (Supplementary Table 13). For the MSI NGS assay, the inter-run and barcode reproducibility study had 100% concordance when comparing the MSI NGS cluster designations (Supplementary Table 13), however four of the twenty samples had disparity in final calling due to inconclusive status related to the cluster boundary (Figure 3B). As with all clinical testing that requires thresholds a transition from a clinical call to an inconclusive status should not be defined as discordant as certain samples will always reside close to the decision boundary defining the inconclusive range. More importantly, when defining reproducibility, an assay should not switch from a MSI-H to MSS or vice versa as this would show a lack in precision in the assay.

#### Between operator variance

Between operator reproducibility was performed on 20 samples across 2 operators, and 2 different runs. Briefly, operator 1 completed libraries for the 20 samples that were sequenced without replication. Operator 2 completed libraries for the same 20 samples that were again sequenced without replication.

For each sample of the inter-operator reproducibility study the number of peaks and mean indel length per loci were calculated to determine the MSI status (Supplementary Table 13). For the MSI NGS assay, the inter-operator reproducibility demonstrated a 100% concordance when comparing the MSI NGS cluster designations between the two replicates of twenty samples, however two samples had individual calls of inconclusive due to centroid distances residing extremely close to the decision boundary (Figure 3C). As previously described, these disparities are regarded as maintaining precision within this assay.

#### Between day variance

Between day reproducibility was evaluated on 20 samples across a single operator, and 2 different days. Briefly, operator 1 completed libraries for the 20 samples that was sequenced without replication on day 2. On a second day, operator 1 completed libraries for the 20 samples that was again sequenced without replication on day 3.

For each sample of the inter-day reproducibility study the number of peaks and mean indel length per loci were calculated to determine the MSI status (Supplementary Table 13). For the MSI NGS assay, the inter-day reproducibility demonstrated a 100% concordance when comparing the MSI NGS cluster designations between the two replicates of twenty samples sequenced on different days (Figure 3D). Three of twenty samples had individual inconclusive calls due to centroid cluster distances close to the decision boundary.

#### Run level controls: NTC, MSI-CTL, and MSS-CTL

The NTC (water) library preparation was included in each run to monitor assay contamination. NTC libraries cannot be sequenced as they gravely effect cluster performance, therefore nM library quantitation (qPCR) and library length (TapeStation, Agilent Technologies, Santa Clara, CA, USA) are assessed and used as contamination threshold metrics. During the validation, the NTC library was generated 14 times as individual replicates producing an average concentration of 4.0 nM (range: 1.6–8.6 nM) with an average library length of 161 bp (range: 147–218) (Supplementary Table 14). As the expected library size of this assay is ~300–350 bp (171 average insert size plus 130 bp of adapter and oligo sequence) and the average library length for the MSI-CTL and MSS-CTL were measured at 321 bp and 327 bp respectively it is evident that the NTC sample is not integrating DNA into the library preparation. Therefore the NTC library thresholds were defined as 8.5 nM (quantitation via qPCR; mean + 2 SDs) and 206 bp (length via TapeStation; mean + 2 SDs) to monitor the assay for gDNA contamination.

The MSI-CTL and MSS-CTL were included in each MSI NGS run as positive and negative run controls. During the validation, the MSI-CTL and MSS-CTL were run a total of 14 times. The correlation of each MSI-CTL and MSS-CTL replicate was calculated by comparing each subsequent run to the first MSI-CTL or MSS-CTL result, with an average correlation of 0.9467 (MSI-CTL) and 0.9265 (MSS-CTL) for number of peaks and 0.9833 (MSI-CTL) and 0.9230 (MSS-CTL) for average indel length demonstrating high correlation and reproducibility at these metrics (Supplementary Table 15). The accurate MSI-H and MSS calls for the MSI-CTL and MSS-CTL, along with other sample level metrics, including percent mapped reads and percent singletons, single-end (non-paired) reads, as well as run level parameters such as Cluster PF%, Total Reads PF, and % ≥Q30 which have been previously defined by Illumina, Inc. [21] are utilized to determine run level QC (Supplementary Tables 16 and 17).

### MSI NGS accuracy

MSI status by NGS and PCR were compared to assess the accuracy of DNA-seq from fifty MSI-H and fifty MSS samples of multiple histologies (Supplementary Table 18). Twenty samples were included per run (10 MSI-H and 10 MSS);for each sample the number of peaks, mean indel length per loci and centroid cluster distances were calculated to determine the MSI status (Supplementary Table 19, QC Metrics: Supplementary Table 17). Although 15 of 100 samples (15%; 8 EEM, 6 CRA, 1 female genital) fell into the inconclusive range predefined during the development of the NGS assay, the overall concordance between MSI NGS and MSI-PCR for the 100 samples was 98% due to two MSI-PCR MSI-H samples being called MSS by MSI NGS (1 EEM and 1 CRA; Supplementary Table 19, highlighted samples). The two false negative cases can be attributed to the fact that these cases are on the edge of the predefined decision boundary of 3.0 between cluster centroid 1 (MSI-H) and cluster centroid 3 (MSS), which is an expected observation when decision boundaries are defined in clinical testing.

The high concordance and absence of any false positive calls of the MSI NGS assay confers the high accuracy of this sequencing workflow and pipeline. From these MSI NGS results, assay sensitivity, specificity, PPV, NPV, and several sequencing level metrics for use as future sample level quality control thresholds were calculated. The sensitivity and specificity of the accuracy study are 96% and 100% respectively with a PPV of 100% and an NPV of 96% with an inconclusive rate of 15% (Table 4).

**Table 4:** Sensitivity and specificity of accuracy study

TP	FP	TN	FN	Inconclusive	Total	Sensitivity	Specificity	PPV	NPV
37	0	46	2	15	100	96%	100%	100%	96%

## DISCUSSION

Traditionally the clinical significance of microsatellite instability status rested on the observations that a subset of MSI-H cancers is associated with Lynch syndrome [22], necessitating further testing, and that MSI-H colorectal cancers have an improved prognosis and do not respond to fluorouracil-based adjuvant chemotherapy [23]. Recently, the discovery that MSI-H tumors respond to PD-L1 checkpoint inhibitor therapy [24–26] has resulted in the first pan-cancer drug indication based on molecular status [27]. The ability to determine MSI status across multiple tumor types is paramount to help identify patients that are likely to respond to CPI therapy while avoiding unnecessary toxicity to patients who are unlikely to respond. We have developed a robust MSI NGS assay that shares comparable specificity and sensitivity to existing gold standard PCR-based methodologies, and which has been CLIA certified and NYS CLEP approved for clinical testing.

The ability to accurately identify MSI status without the need of matched normal tissue, a major hurdle in clinical testing, relies on the use of our MSI caller which utilizes 29 loci targeting homopolymer tandem repeat regions within the genome, integrating both mean indel length and number of unique indel peaks at each loci to define Euclidian distance and its association to cluster centroids to define MSI status. Utilizing a gold standard sample set that has defined MSI status using PCR-based clinical testing as the training and test set we developed the MSI caller which showed 100% sensitivity and 100% specificity in both groups with an inconclusive status in reporting of approximately 10%. As described, the inconclusive range is determined by a defined Euclidean distance between MSI-H cluster 1 and MSS cluster 3. The integration of an inconclusive status helps to protect against false positive and false negative reporting which may lead to detrimental pharmacotherapy. To this end, a sample which resides in the inconclusive range will be reported clinically as inconclusive, which would indicate therapy should not be pursued in this small subset of patients.

Utilizing the MSI Caller algorithm and a defined set of gold-standard clinical samples, where MSI status has previously been reported using a PCR based clinical assay, we have carried out an analytical validation of a MSI NGS assay that can be utilized to determine MSI status in solid tumors for stratification of treatment to CPI therapy. As part of the analytical validation and assay robustness, precision and accuracy were determined. The MSI NGS assays robustness, or measure of the assays capacity to remain unaffected by small variations in procedural parameters, defined a tolerance to a minimum DNA input of 5 ng (although standard input is 20 ng), the sensitivity to detect genomic instability in tumors from 10%–100% neoplastic nuclei content, and the ability to run various batch sizes from 5 to 40 samples per run all of which are critical to help mitigate the vast sample variances identified within the clinical laboratory workflow. Precision of the MSI NGS assay was determined through a series of reproducibility experiments to ensure the test’s ability to make concordant calling across intra-run, inter-run, inter-operator, inter-day, and inter-barcode variance. Furthermore, during the reproducibility studies, the utility and thresholds associated with a common set of control samples (NTC, MSI-CTL, and MSS-CTL) which are to be included in every clinical run were defined. To measure accuracy of the MSI NGS assay 100 gold-standard solid tumor samples previously tested using our NYS CLEP approved MSI-PCR assay (Project ID:709) were utilized. The reported sensitivity and specificity of the accuracy study are 96% and 100% respectively with a PPV of 100% and an NPV of 96% with an inconclusive rate of 15%.

While the PCR-based Bethesda markers were designed for MSI profiling in CRC, the large number of target regions included in the MSI NGS assay allows for increased confidence in pan-cancer testing without the requirement for matched normal tissue, a key component to the significant advantage over PCR. Prior to MSI NGS testing in our laboratory performing MSI in a pan-cancer setting up to 40% of clinical orders lacked matched normal tissue and required subsequent requests for alternate blocks or blood to perform MSI-PCR. Of the cases where alternate material were requested slightly less than one-half yielded matched normal material for testing with an excessive wait time of typically 10 to12 days leading to delayed reporting and an overall inability to complete 20% of all clinical requests for MSI testing. Since the activation of the MSI NGS assay within the clinical laboratory the need to request and wait for matched normal tissue has been alleviated and all cases that meet the requirements set during the analytical validation can be tested, greatly reducing turnaround time and failure rates within the lab.

Overall, we have developed the first NYS-CLEP approved, analytically validated MSI assay that requires minimum input of tumor DNA without the need for matched normal and is histology agnostic. Although it has an equivalent cost and turnaround times to MSI-PCR, it is much more scalable at a high-volume laboratory. Although this assay is NGS-based, the workflow is performed similarly to a single analyte test and can be efficiently and inexpensively integrated into any molecular diagnostic laboratory.

## MATERIALS AND METHODS

### Specimens

Samples were procured with informed patient consent under an institutional banking policy (IRB Protocol I115707) and the study was approved by the internal review board at Roswell Park Comprehensive Cancer Center (IRB Protocol # BRD 073116). For assay validation, 100 fixed paraffin embedded (FFPE) human clinical specimens collected from 2009–2017 and a subset of matched normal tissues from colorectal cancer (73 cases), endometrioid carcinoma (18), uterine (4), small intestine (2), prostate (1), stomach adenocarcinoma (1), and female genital (1) cancer patients stored at the OmniSeq, Inc. (Buffalo, NY, USA) and Roswell Park Comprehensive Cancer Center (Buffalo, NY, USA) remnant tissue biobanks were used to evaluate the performance of the MSI NGS assay. Two human cell lines, non-small cell lung cancer (NSCLC) HCC-78 cells (DSMZ, Braunschweig, Germany), and colorectal cancer (CRC) HCT-116 cells (ATCC, Manassas, VA, USA) processed as FFPE blocks were also used for development and as internal run controls.

### Tissue QC and extraction

A hematoxylin and eosin (H&E) stained tumor and normal tissue sections were reviewed by a board-certified anatomical pathologist to establish tissue QC parameters. Criteria for neoplastic testing was ≥ 2 mm^2^ of tumor surface area per slide, with tumor cellularity ≥ 10% and necrosis ≤ 50%. For level of detection studies, non-malignant tissue was also identified for isolation and reviewed to exclude any neoplastic tissue. Genomic DNA was extracted from areas identified by the pathologist using 3–5 unstained slides with the truXTRAC FFPE extraction kit (Covaris, Inc., Woburn, MA, USA), as described previously. DNA was eluted in 100 µL water, and yield was determined by the Qubit DNA HS Assay (Thermo Fisher Scientific, Waltham, MA, USA), as per manufacturer’s recommendation. To ensure adequate library preparation, a predefined yield of 20 ng DNA was used.

### Run controls

To establish thresholds and daily QC parameters, run controls were identified and included in each library preparation batch and NGS run. They included both MSI positive (HCT-116) and MSS (HCC-78) controls, as well as a no template control (NTC, water). Positive controls provide templates for all targets for MSI-H interpretations, while negative controls monitor assay specificity. The NTC is used to monitor assay contamination.

### Library preparation and NGS

MSI NGS libraries were prepared from 20 ng DNA using the TruSeq Custom Amplicon Low Input Kit (Illumina, San Diego, CA, USA). The panel content is detailed in section “Results: Assay development”. Following hybridization, oligos were extended, ligated, and unique indexes were added. Libraries were amplified, purified, quantitated and normalized to 4 nM. Up to 40 equimolar libraries were pooled, denatured and further diluted to 7 pM. Pooled libraries were sequenced on a MiSeq (Illumina) sequencer using a 300 cycle paired end sequencing kit to obtain 500X mean depth per sample.

### Accuracy studies

MSI NGS accuracy was evaluated by comparison with a PCR based NYS CLEP-approved assay for all samples. For gold standard PCR analysis, 4 sets of fluorescently-labeled primers were used for amplification of five markers (BAT-25, BAT-26, D2S123, D5S346, and D17S250). Internal lane size standards added to PCR products assured accurate sizing of alleles and to adjust for run-to-run variation. PCR products were separated by capillary electrophoresis using ABI PRISM 3500xl Genetic Analyzer and output data was analyzed with GeneMapper Software 5 (both Applied Biosystems, Foster City, CA, USA).

### NGS analysis pipeline and QC

QC metrics were established and defined at the run and sample level to ensure high quality results and to monitor any run to run variance or long term drift (Supplementary Table 20). Sequencing data from the Illumina platform were first processed using custom bioinformatics pipeline for reference mapping and indel calling, during which validation-defined quality control (QC) specifications for depth of coverage were used as acceptance criteria. To ensure high quality results, a QC system was developed based on NGS data generated at validation. The QC criteria were established for several metrics at the run, sample, and run control thresholds, with defined values to accept or reject one or more aspects of sequencing. Likewise, specific QC metrics were monitored over time to detect any potential long-term assay drift. Quality filters were used at the amplicon level to remove counts below the threshold for detection, and at the base-pair level for low-quality indel calls.

A custom MSI NGS caller and pipeline was developed to predict the MSI status from NGS data utilizing a training set (*n* = 94; MSI-H = 22, MSS = 24) and test set (*n* = 48; MS--H = 22, MSS = 24) of gold-standard MSI-PCR samples collected from the clinical laboratory archive (see Results: Development of MSI NGS Caller). In general, the pipeline was designed to read “.fastq” files of all samples from a sequencing run and conduct sequence alignment, variant calling, indel extraction, indel length and number of peaks calculation and MSI prediction (Supplementary Figure 1). Specifically, in the first part of the algorithm fastq file of each sample within a run was aligned to human genome (hg19) using bwa resulting in SAM genome alignment file which was further sorted and converted to BAM format and indexed for faster processing. This was followed by the crucial variant calling step wherein, all the aligned reads within a sample specific indexed BAM file were then used for custom variant calling (TNscope v201711.02, Sentieon Inc., Mountain View, CA, USA) to generate VCF (variant call file) files. This was followed by extraction of indel calls from the VCF files which were then used to calculate number of peaks (number of unique indels at each loci) and average indel length for 29 loci identified in the MSI call development as follows: For each locus X, Average indel ${\text{Length}}_{\left(\text{L}\right)}=\frac{\sum_{i=1}^{n}allelic\_length_{i}}{Todal\;Number\;of\;alleles\;at\;loci"X"}$ and For each locus X, Number of Peaks (nPeaks_(X)_) = *Total number of alleles at loci “X”* Finally, MSI classification was then performed using these 58 features where, Euclidean distance was calculated between each new sample and the centroid of original training kmeans clusters. The closest cluster by Euclidean distance was then used to assign a MSI NGS prediction for the sample (Supplementary Figure 2).

### Statistical analysis

Principal component analysis (FactoMineR v1.41 in R v3.4.2) was performed on 58 features to visualize the separation between MSI-H and MSS/Normal samples. To develop a predictive model, Kmeans clustering (kmeans stats package in R v3.4.2) was performed to identify three centroids (k = 3) that represent two MSI-H clusters and a combined MSS/Normal cluster. This led to an intuitive and simplistic method of assigning future samples to each of the original clusters using simple Euclidean distance measure. Correlation measure used throughout the study is Pearson’s correlation coefficient denoted as “r”. Standard performance metrics are defined as Sensitivity $\left(\frac{TP}{\left[TP+FN\right]}\right)$, specificity $\left(\frac{TN}{\left[TN+FP\right]}\right)$, PPV $\left(\frac{TP}{\left[TP+FP\right]}\right)$, NPV $\left(\frac{TP}{\left(TN+FN\right)}\right)$ and accuracy $\left(\frac{TP+TN}{\left(TP+FP+TN+FN\right)}\right)$.

## SUPPLEMENTARY MATERIALS

























## Abbreviations

NGS: next-generation sequencing
