# Supplementary material for: Development and analytical validation of a next-generation sequencing based microsatellite instability (MSI) assay
Source: Oncotarget. 2019 Aug 27;10(50):5181–93. doi: 10.18632/oncotarget.27142 (PMC6718258; doi:10.18632/oncotarget.27142)
Supplement: Supplementary file 1 [file oncotarget-10-5181-s001.pdf]

Development and analytical validation of a next-generation sequencing based microsatellite instability (MSI) assay

SUPPLEMENTARY MATERIALS

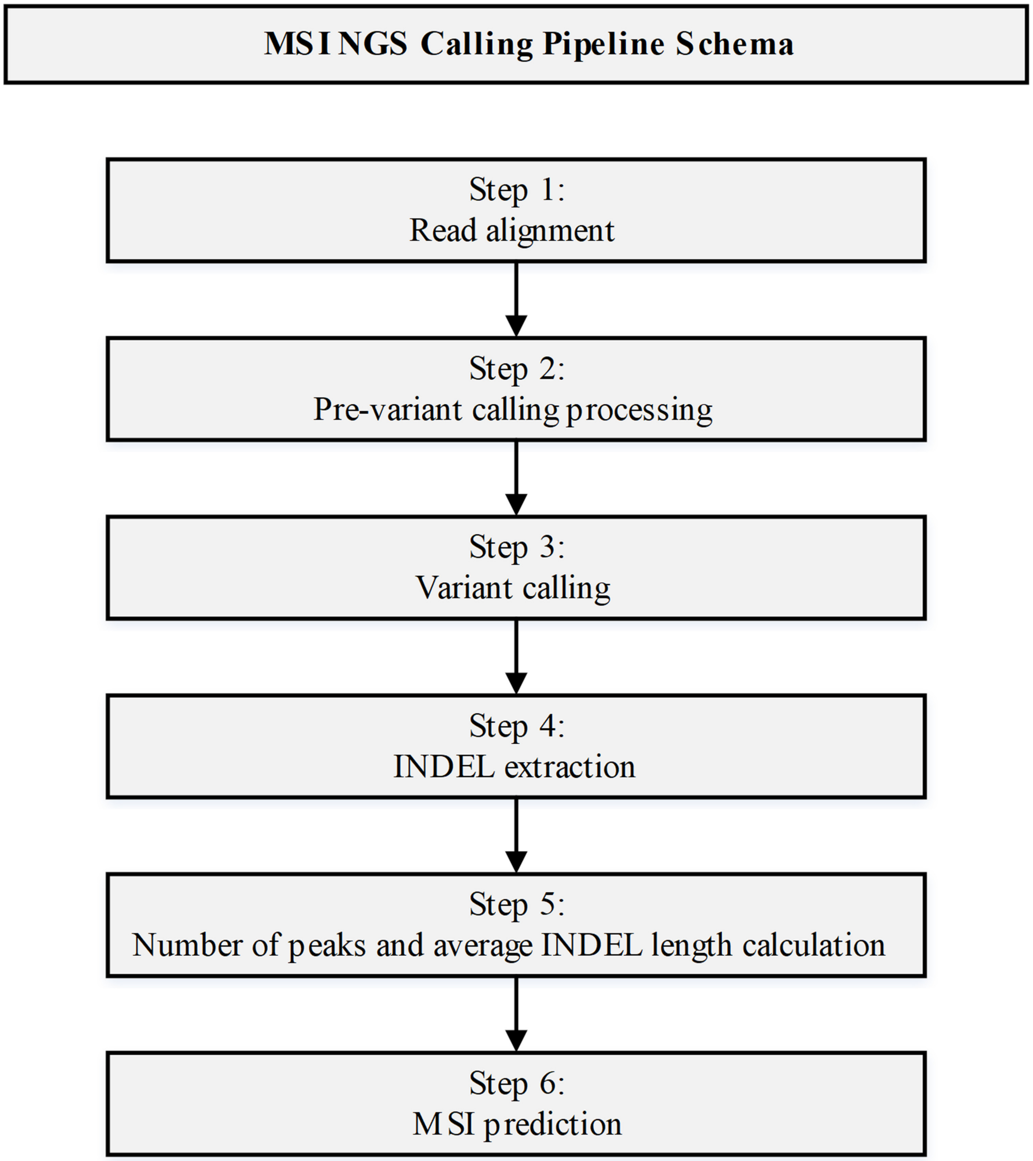

Supplementary Figure 1: Custom bioinformatics pipeline for identification of microsatellite instability using NGS. The pipeline was designed to read ".fastq" files of all samples from a sequencing run and conduct sequence alignment, variant calling, indel extraction, indel length and number of peaks calculation and MSI prediction.

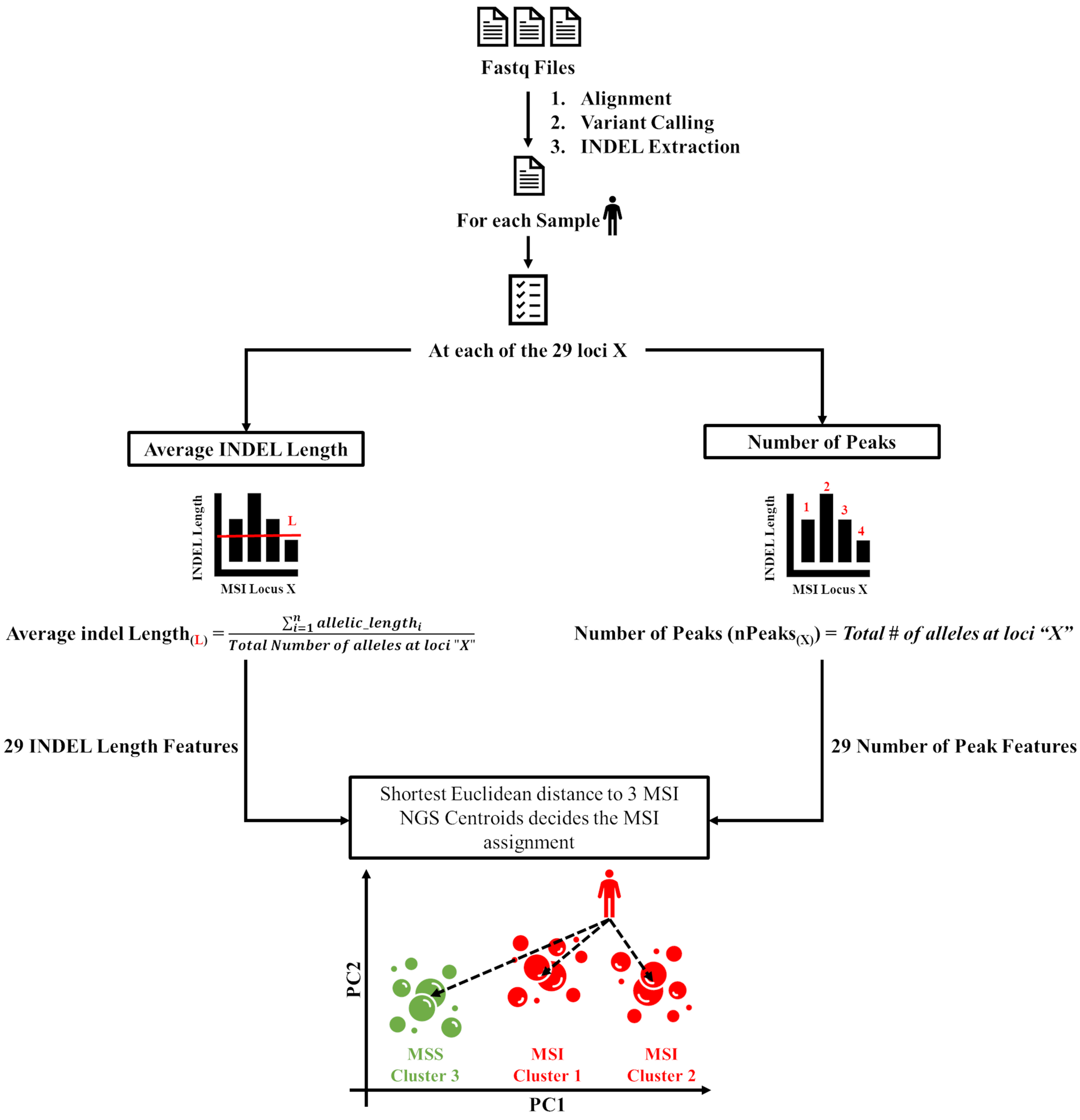

Supplementary Figure 2: A representative flow chart depicting the overview of MSI NGS prediction method.

Supplementary Table 1: TCGA filtered INDEL calls for MSI NGS panel development

CHR	POS	REF	ALT	ALT LEN	VAR TYPE	Selected	Proportion of MSI Negative	Proportion of MSI Positive	Fisher's Exact Test, *p*
chr2	62063093	T	AAAAA	5	DEL	Y	0	7	4.91207E-07
chr6	111329221	C	TTTTT	5	DEL	Y	0	7	4.91207E-07
chr7	102727051	C	TTTTT	5	DEL	Y	0	7	4.91207E-07
chr10	112360315	G	TTTTT	5	DEL	Y	0	7	4.91207E-07
chr11	14539315	T	AAAAA	5	DEL	Y	0	7	4.91207E-07
chr12	112893675	G	TTTTT	5	DEL	Y	0	7	4.91207E-07
chr14	66964769	G	TTTTT	5	DEL	Y	0	7	4.91207E-07
chr16	11064865	A	TTTTTT	6	DEL	Y	0	7	4.91207E-07
chr17	19314917	C	TTTTT	5	DEL	Y	0	7	4.91207E-07
chr19	21561408	C	TTTTT	5	DEL	Y	0	7	4.91207E-07
chrX	48243983	C	TTTTT	5	DEL	Y	0	7	4.91207E-07
chr3	43607224	C	AAAAA	5	DEL	Y	1	7	3.92966E-06
chr12	8286096	A	TTTTT	5	DEL	Y	1	7	3.92966E-06
chr1	7913184	C	AAAAA	5	DEL	Y	0	6	1.1789E-05
chr1	16388875	G	CCCCC	5	DEL	Y	0	6	1.1789E-05
chr1	74901739	C	TTTTT	5	DEL	Y	0	6	1.1789E-05
chr1	200312546	C	AAAAA	5	DEL	Y	0	6	1.1789E-05
chr1	231094050	G	AAAAA	5	DEL	Y	0	6	1.1789E-05
chr2	91872178	A	TTTTTT	6	DEL	Y	0	6	1.1789E-05
chr2	91872178	A	TTTTTTT	7	DEL	Y	0	6	1.1789E-05
chr2	108479622	C	TTTTT	5	DEL	Y	0	6	1.1789E-05
chr2	203676382	C	AAAAA	5	DEL	Y	0	6	1.1789E-05
chr2	203676382	C	AAAAAA	6	DEL	Y	0	6	1.1789E-05
chr2	234178624	C	TTTTT	5	DEL	Y	0	6	1.1789E-05
chr3	129168854	C	TTTTT	5	DEL	Y	0	6	1.1789E-05
chr3	132221076	C	TTTTT	5	DEL	Y	0	6	1.1789E-05
chr4	38134423	G	TTTTT	5	DEL	Y	0	6	1.1789E-05
chr4	47856957	C	AAAAA	5	DEL	Y	0	6	1.1789E-05
chr5	70888665	G	TTTTT	5	DEL	Y	0	6	1.1789E-05
chr5	96093391	G	TTTTT	5	DEL	Y	0	6	1.1789E-05
chr5	134106506	C	AAAAA	5	DEL	Y	0	6	1.1789E-05
chr6	84061864	G	AAAAA	5	DEL	Y	0	6	1.1789E-05
chr8	59060725	G	TTTTT	5	DEL	Y	0	6	1.1789E-05
chr9	77550305	C	AAAAA	5	DEL	Y	0	6	1.1789E-05
chr9	86617961	C	TTTTT	5	DEL	Y	0	6	1.1789E-05
chr12	89853387	C	AAAAA	5	DEL	Y	0	6	1.1789E-05
chr14	75181481	C	AAAAA	5	DEL	Y	0	6	1.1789E-05
chr15	56243792	T	AAAAA	5	DEL	Y	0	6	1.1789E-05
chr16	11064865	A	TTTTT	5	DEL	Y	0	6	1.1789E-05
chr16	75674252	T	AAAAAA	6	DEL	Y	0	6	1.1789E-05
chr17	36374963	T	AAAAA	5	DEL	Y	0	6	1.1789E-05
chr18	649879	C	TTTTT	5	DEL	Y	0	6	1.1789E-05
chr19	21561408	C	TTTTTT	6	DEL	Y	0	6	1.1789E-05
chr19	21561408	C	TTTTTTT	7	DEL	Y	0	6	1.1789E-05
chr20	3298881	C	AAAAAA	6	DEL	Y	0	6	1.1789E-05
chr22	44083327	G	TTTTTT	6	DEL	Y	0	6	1.1789E-05
chrX	129630732	T	AAAAA	5	DEL	Y	0	6	1.1789E-05

Supplementary Table 2: MSI NGS peak number identified in training set. See Supplementary_ Table_2

Supplementary Table 3: MSI NGS loci prevelance in training set. See Supplementary_Table_3

Supplementary Table 4: MSI NGS centroid values for each locus. See Supplementary_Table_4

Supplementary Table 5: MSI NGS prediction in training set

Sample Name	Tissue Type	Prediction Cluster	MSI NGS	MSI PCR	Cluster 1	Cluster 2	Cluster 3	Difference (Cluster 3 and Cluster 1)
RS-2774587	CRC	1	MSI-H	MSI-H	4.835573058	12.31609386	5.363296362	–0.527723303
RS-2774589	CRC	1	MSI-H	MSI-H	6.440252143	7.260850683	12.70196987	–6.261717728
RS-2774606	CRC	1	MSI-H	MSI-H	4.576201305	9.95291329	8.510299989	–3.934098684
RS-2774607	endometrial endometrioid carcinoma	1	MSI-H	MSI-H	5.184442643	6.838563909	11.81155411	–6.627111472
RS-2774608	endometrial endometrioid carcinoma	1	MSI-H	MSI-H	4.25612464	11.75545541	6.325832885	–2.069708245
RS-2774617	endometrial endometrioid carcinoma	1	MSI-H	MSI-H	5.103702614	10.39349847	8.293174435	–3.189471821
RS-2774619	endometrial endometrioid carcinoma	1	MSI-H	MSI-H	4.64416444	10.55630954	8.478582924	–3.834418484
RS-2774622	endometrial endometrioid carcinoma	1	MSI-H	MSI-H	4.7274224	7.474941815	10.90817491	–6.180752514
RS-2774623	endometrial endometrioid carcinoma	1	MSI-H	MSI-H	4.188269876	11.28216122	6.998993768	–2.810723891
RS-2774585	CRC	2	MSI-H	MSI-H	7.470995279	5.063026601	14.17213793	–6.701142654
RS-2774588	CRC	2	MSI-H	MSI-H	11.39921236	6.244021924	18.08750673	–6.688294373
RS-2774591	CRC	2	MSI-H	MSI-H	11.21346738	5.451079785	17.78343772	–6.569970342
RS-2774592	CRC	2	MSI-H	MSI-H	7.242926136	6.315510611	14.06792996	–6.825003828
RS-2774601	CRC	2	MSI-H	MSI-H	12.46181411	5.306446321	19.58624497	–7.124430851
RS-2774602	CRC	2	MSI-H	MSI-H	10.94840166	4.509762888	17.68877401	–6.740372356
RS-2774604	CRC	2	MSI-H	MSI-H	9.882771489	4.109701889	16.89284147	–7.010069985
RS-2774605	CRC	2	MSI-H	MSI-H	10.54970316	5.699110718	16.91761036	–6.367907191
RS-2774618	CRC	2	MSI-H	MSI-H	11.27156501	5.33534894	18.23691279	–6.965347773
RS-2774621	CRC	2	MSI-H	MSI-H	8.438478261	5.608506241	14.89747029	–6.458992029
RS-2774590	stomach adenocarcinoma	2	MSI-H	MSI-H	9.760559566	5.230741893	16.08438031	–6.323820742
**RS-2774620**	CRC	3	**MSS**	**MSI-H**	5.990155449	13.69119603	4.082762283	1.907393165
RS-2774633	CRC	3	MSS	MSS	7.26326176	14.87684421	4.023329225	3.239932534
RS-2774635	CRC	3	MSS	MSS	9.082279676	17.31020819	3.211384733	5.870894943
RS-2774636	CRC	3	MSS	MSS	9.08925816	16.98843942	3.436848372	5.652409789
RS-2774637	CRC	3	MSS	MSS	8.291331424	15.90923716	5.566203222	2.725128202
RS-2774638	CRC	3	MSS	MSS	10.4569478	18.39947052	4.880597706	5.576350095
RS-2774639	CRC	3	MSS	MSS	9.527773375	17.08387243	5.214356507	4.313416868
RS-2774640	CRC	3	MSS	MSS	10.41695437	18.13683369	4.426673359	5.990281015
RS-2774649	CRC	3	MSS	MSS	7.649347544	15.33881194	3.594249033	4.055098511
RS-2774651	CRC	3	MSS	MSS	8.959829651	16.56420867	4.149624156	4.810205494
RS-2774652	CRC	3	MSS	MSS	8.390569571	16.23562533	3.94392846	4.446641111
RS-2774653	CRC	3	MSS	MSS	8.352232408	16.36841789	3.392713983	4.959518425
RS-2774654	CRC	3	MSS	MSS	9.544925699	17.43988284	4.603937847	4.940987852
RS-2774655	CRC	3	MSS	MSS	8.285872423	15.84275058	4.659831833	3.62604059
RS-2774663	CRC	3	MSS	MSS	9.697061418	17.39110013	4.335416844	5.361644574
RS-2774664	CRC	3	MSS	MSS	8.381345704	16.02327933	3.740496576	4.640849128
RS-2774665	CRC	3	MSS	MSS	7.542433607	15.4986641	3.139029609	4.403403999
RS-2774666	CRC	3	MSS	MSS	7.85441762	15.53540397	3.264374347	4.590043274
RS-2774667	CRC	3	MSS	MSS	10.15264063	17.83439564	4.815512909	5.337127721
RS-2774668	CRC	3	MSS	MSS	9.46055022	17.33226467	3.951237479	5.509312741
RS-2774669	CRC	3	MSS	MSS	8.963937777	16.38971654	4.511059736	4.452878041
RS-2774670	CRC	3	MSS	MSS	7.614443128	15.75587351	2.936413691	4.678029437
RS-2774624	CRC	3	MSS	MSS	7.632272607	15.75789558	3.193738313	4.438534294
**RS-2774603**	endometrial endometrioid carcinoma	3	**MSS**	**MSI-H**	7.044110867	14.25748593	4.684243801	2.359867067
RS-2774586	endometrial endometrioid carcinoma	3	MSS	MSS	8.123111514	15.56195874	4.856907509	3.266204005

RS-2774634	endometrial endometrioid carcinoma	3	MSS	MSS	6.402380712	14.36883764	3.56554565	2.836835062
RS-2774650	endometrial endometrioid carcinoma	3	MSS	MSS	8.049829607	15.89583879	3.319946263	4.729883344
RS-2774593	Normal	3	MSS	Normal	9.037988881	16.39429444	4.712681623	4.325307258
RS-2774594	Normal	3	MSS	Normal	8.474956476	16.25044909	4.307754235	4.167202241
RS-2774595	Normal	3	MSS	Normal	8.636660621	16.76162568	3.250278093	5.386382528
RS-2774596	Normal	3	MSS	Normal	7.52627847	15.83898064	2.645751848	4.880526621
RS-2774597	Normal	3	MSS	Normal	7.3838173	15.16519258	3.44735561	3.93646169
RS-2774598	Normal	3	MSS	Normal	8.948277788	16.83764902	3.874595044	5.073682744
RS-2774599	Normal	3	MSS	Normal	8.186021174	16.02901966	2.956504358	5.229516816
RS-2774600	Normal	3	MSS	Normal	9.163962076	17.18198796	4.447064916	4.71689716
RS-2774609	Normal	3	MSS	Normal	8.307516045	15.91182854	4.378848397	3.928667648
RS-2774610	Normal	3	MSS	Normal	11.20617979	18.87381017	5.351771704	5.854408086
RS-2774611	Normal	3	MSS	Normal	8.884167657	16.60491166	4.15518779	4.728979867
RS-2774612	Normal	3	MSS	Normal	10.9136231	18.79492126	5.254743179	5.658879922
RS-2774613	Normal	3	MSS	Normal	10.06825131	17.91390957	3.964977724	6.103273587
RS-2774614	Normal	3	MSS	Normal	8.144820307	15.76798784	4.900014791	3.244805516
RS-2774615	Normal	3	MSS	Normal	9.514636093	17.44084197	4.478705932	5.035930161
RS-2774616	Normal	3	MSS	Normal	7.373045424	15.58226099	3.270195585	4.102849839
RS-2774625	Normal	3	MSS	Normal	7.81618978	15.2043099	4.530136526	3.286053255
RS-2774626	Normal	3	MSS	Normal	6.288134985	14.17422524	3.514728894	2.773406091
RS-2774627	Normal	3	MSS	Normal	8.362500395	16.03527754	4.153673497	4.208826897
RS-2774628	Normal	3	MSS	Normal	8.194076121	16.05825893	3.319262879	4.874813242
RS-2774629	Normal	3	MSS	Normal	8.978947544	17.07724059	3.861793885	5.117153659
RS-2774630	Normal	3	MSS	Normal	9.983685438	17.41304731	5.5607025	4.422982938
RS-2774631	Normal	3	MSS	Normal	8.641716215	16.63072758	2.944926097	5.696790118
RS-2774632	Normal	3	MSS	Normal	7.444171711	15.21608201	3.482182075	3.961989635
RS-2774641	Normal	3	MSS	Normal	8.487374561	16.03633038	3.965932362	4.5214422
RS-2774642	Normal	3	MSS	Normal	9.669498547	17.23773597	5.06112858	4.608369967
RS-2774643	Normal	3	MSS	Normal	10.859552	18.43617171	5.599758824	5.259793176
RS-2774644	Normal	3	MSS	Normal	9.080479264	16.96223639	3.830693347	5.249785917
RS-2774645	Normal	3	MSS	Normal	9.220418635	16.88020999	5.641751728	3.578666907
RS-2774646	Normal	3	MSS	Normal	8.674919223	16.73441812	4.319159907	4.355759317
RS-2774647	Normal	3	MSS	Normal	9.790768781	17.74969065	3.559715747	6.231053033
RS-2774648	Normal	3	MSS	Normal	9.458275491	17.30963475	3.894748549	5.563526942
RS-2774656	Normal	3	MSS	Normal	10.2612038	16.93609401	6.362244389	3.898959414
RS-2774657	Normal	3	MSS	Normal	8.127412818	15.93971789	4.266251365	3.861161453
RS-2774658	Normal	3	MSS	Normal	9.21892691	16.3207977	6.099355658	3.119571251
RS-2774659	Normal	3	MSS	Normal	8.0197359	16.11719467	3.330750713	4.688985187
RS-2774660	Normal	3	MSS	Normal	7.2722113	15.10732479	3.785335827	3.486875474
RS-2774661	Normal	3	MSS	Normal	7.566258168	15.8180344	3.337378924	4.228879244
RS-2774662	Normal	3	MSS	Normal	10.2137994	17.94531396	5.136452254	5.077347148
RS-2774671	Normal	3	MSS	Normal	8.884946272	16.4735685	4.333955667	4.550990605
RS-2774672	Normal	3	MSS	Normal	9.443929592	17.46592032	3.72489962	5.719029972
RS-2774673	Normal	3	MSS	Normal	6.807363211	15.09201944	2.29973341	4.507629801
RS-2774674	Normal	3	MSS	Normal	7.773838695	15.64414528	3.249734584	4.524104111
RS-2774675	Normal	3	MSS	Normal	9.173541057	17.19983566	3.487861294	5.685679763
RS-2774676	Normal	3	MSS	Normal	7.298938089	15.24965177	2.560566773	4.738371316
RS-2774677	Normal	3	MSS	Normal	12.5064456	20.01914496	6.379604952	6.126840648
RS-2774678	Normal	3	MSS	Normal	8.956577208	17.08200226	3.260809579	5.695767629

Supplementary Table 6: MSI NGS prediction in validation set. See Supplementary_Table_6

Supplementary Table 7: NGS detection of difference in number of peaks at BAT25 marker location for BAT25 unstable cases by gold standard PCR

Tumor	Matched Normal	BAT25-NGS-nPeaks-Tumor	BAT25-NGS-nPeaks-Normal	NGS Peak Difference	BAT25-PCR-Peaks-Tumor	BAT25-PCR-Peaks-Normal	PCR Peak Difference
RS-2774601	RS-2774609	19	12	7	3	1	2
RS-2774590	RS-2774598	17	10	7	2	1	1
RS-2774602	RS-2774610	17	10	7	2	1	1
RS-2774592	RS-2774600	16	10	6	2	1	1
RS-2774605	RS-2774613	17	11	6	2	1	1
RS-2774621	RS-2774629	18	12	6	2	1	1
RS-2774591	RS-2774599	15	11	4	3	1	2
RS-2774607	RS-2774615	15	11	4	3	1	2
RS-2774588	RS-2774596	16	12	4	3	1	2
RS-2774622	RS-2774630	15	11	4	2	1	1
RS-2774618	RS-2774626	17	13	4	2	1	1
RS-2774619	RS-2774627	14	11	3	2	1	1
RS-2774620	RS-2774628	12	10	2	2	1	1
RS-2774617	RS-2774625	13	11	2	4	3	1
RS-2774589	RS-2774597	14	12	2	2	1	1
RS-2774624	RS-2774632	11	12	1	2	1	1
RS-2774608	RS-2774616	13	12	1	3	2	1
RS-2774603	RS-2774611	12	12	0	2	1	1

Supplementary Table 8: NGS detection of difference in number of peaks at BAT26 marker location for BAT26 Unstable cases by gold standard PCR

Tumor	Matched Normal	BAT26-NGS-nPeaks-Tumor	BAT26-NGS-nPeaks-Normal	NGS Peak Difference	BAT26-PCR-Peaks-Tumor	BAT26-PCR-Peaks-Normal	PCR Peak Difference
RS-2774588	RS-2774596	15	7	8	2	1	1
RS-2774591	RS-2774599	13	7	6	2	1	1
RS-2774601	RS-2774609	13	7	6	2	1	1
RS-2774602	RS-2774610	13	7	6	2	1	1
RS-2774605	RS-2774613	13	7	6	2	1	1
RS-2774590	RS-2774598	13	8	5	2	1	1
RS-2774592	RS-2774600	11	7	4	2	1	1
RS-2774617	RS-2774625	11	7	4	3	2	1
RS-2774607	RS-2774615	9	6	3	3	1	2
RS-2774621	RS-2774629	9	6	3	2	1	1
RS-2774589	RS-2774597	10	7	3	2	1	1
RS-2774608	RS-2774616	10	7	3	4	3	1
RS-2774619	RS-2774627	10	7	3	2	1	1
RS-2774618	RS-2774626	10	8	2	3	1	2
RS-2774623	RS-2774631	8	7	1	3	1	2
RS-2774622	RS-2774630	9	8	1	3	1	2
RS-2774624	RS-2774632	7	8	1	2	1	1
RS-2774603	RS-2774611	8	7	1	2	1	1
RS-2774620	RS-2774628	8	7	1	2	1	1
RS-2774606	RS-2774614	8	8	0	2	1	1

Supplementary Table 9: Effect of percent tumor nuclei on MSI NGS calling. See Supplementary_Table_9

Supplementary Table 10: Effect of DNA (ng) Input on MSI NGS calling. See Supplementary_Table_10

Supplementary Table 11: Effect of batch size on MSI NGS calling. See Supplementary_Table_11

Supplementary Table 12: Sample level quantitation and QC data for reproducibility study

Sample ID	Run	Library Yield	Depth of Coverage	mapped_reads_pct	singletons_pct
RD-5304-1	RP1	85.6	2209	98.0023	0.279145
RD-5304-2	RP1	60.2	2020	96.3624	0.392017
RD-5304-3	RP1	75.9	2305	96.3624	0.392017
RD-5311-1	RP1	85.4	2114	88.6036	0.947926
RD-5311-2	RP1	109.9	1848	96.4373	0.357335
RD-5311-3	RP1	43.4	1459	96.4373	0.357335
RD-5312-1	RP1	176.1	2219	93.6922	0.656078
RD-5312-2	RP1	179.4	2183	93.6922	0.656078
RD-5312-3	RP1	138.1	2827	93.6922	0.656078
RD-5313-1	RP1	190.3	2672	94.3748	0.500055
RD-5313-2	RP1	178.8	2514	94.3748	0.500055
RD-5313-3	RP1	178.3	2612	94.3748	0.500055
RD-5345-1	RP1	55.2	2406	93.6922	0.656078
RD-5345-2	RP1	57.2	2080	92.3219	0.59737
RD-5345-3	RP1	19.3	952	92.3219	0.59737
RD-5346-1	RP1	82.9	3618	80.9851	1.5201
RD-5346-2	RP1	138.6	2971	92.9183	0.578603
RD-5346-3	RP1	156.9	2878	92.9183	0.578603
RD-5279-R1-T1	RP2	55.9	2422	93.8072	0.562055
RD-5280-R1-T1	RP2	24.2	1914	95.6796	0.774148
RD-5281-R1-T1	RP2	26.5	2056	92.8141	0.607963
RD-5282-R1-T1	RP2	56.7	2510	96.9827	0.795453
RD-5283-R1-T1	RP2	41.0	2400	92.9368	0.4015
RD-5284-R1-T1	RP2	74.2	2449	95.6963	0.464343
RD-5285-R1-T1	RP2	39.9	2033	94.9346	0.524148
RD-5286-R1-T1	RP2	115.9	3103	82.5901	0.538592
RD-5287-R1-T1	RP2	18.3	1855	95.581	0.633818
RD-5288-R1-T1	RP2	59.2	2497	93.6543	0.434018
RD-5289-R1-T1	RP2	87.1	2445	95.6963	0.527724
RD-5290-R1-T1	RP2	88.9	2322	96.626	0.958923
RD-5291-R1-T1	RP2	81.3	2656	90.8441	0.466897
RD-5292-R1-T1	RP2	102.3	2351	95.9487	0.703172
RD-5293-R1-T1	RP2	37.3	2142	96.9315	0.613436
RD-5294-R1-T1	RP2	68.6	2443	95.4313	0.701861
RD-5295-R1-T1	RP2	72.9	2200	93.2658	0.630016
RD-5296-R1-T1	RP2	61.9	2172	94.039	0.606404
RD-5297-R1-T1	RP2	72.3	2256	94.4695	1.03252
RD-5298-R1-T1	RP2	49.2	1883	90.0165	1.92836
RD-5279-R2-T2	RP3	87.7	2678	93.5683	0.503304
RD-5280-R2-T2	RP3	58.4	2350	93.4865	0.51102
RD-5281-R2-T2	RP3	66.7	2124	92.4202	0.659091
RD-5282-R2-T2	RP3	87.0	2806	91.4026	0.738055
RD-5283-R2-T2	RP3	60.2	2209	93.7032	0.480255
RD-5284-R2-T2	RP3	110.9	2108	88.3498	0.851301

RD-5285-R2-T2	RP3	28.9	1700	93.056	0.640527
RD-5286-R2-T2	RP3	120.9	2778	92.9624	0.546469
RD-5287-R2-T2	RP3	68.7	1864	91.835	0.618407
RD-5288-R2-T2	RP3	101.8	2130	94.391	0.506297
RD-5289-R2-T2	RP3	138.7	2307	94.5516	0.510076
RD-5290-R2-T2	RP3	157.0	2521	94.3438	0.526232
RD-5291-R2-T2	RP3	132.8	2398	93.2064	0.580682
RD-5292-R2-T2	RP3	166.2	2424	94.1598	0.511901
RD-5293-R2-T2	RP3	77.8	2000	91.3964	0.783085
RD-5294-R2-T2	RP3	108.1	2130	90.7282	0.74809
RD-5295-R2-T2	RP3	72.9	2196	93.3636	0.564241
RD-5296-R2-T2	RP3	109.9	2274	95.1988	0.405725
RD-5297-R2-T2	RP3	92.7	2135	93.0392	0.620059
RD-5298-R2-T2	RP3	56.1	1649	77.4681	1.90183
RD-5279-R3-T1	RP4	127.1	2964	90.0065	0.841184
RD-5280-R3-T1	RP4	131.7	2196	89.7662	0.873407
RD-5281-R3-T1	RP4	116.0	2234	87.8493	1.21544
RD-5282-R3-T1	RP4	95.6	2101	83.7608	1.48475
RD-5283-R3-T1	RP4	69.3	1732	81.2577	1.53329
RD-5284-R3-T1	RP4	181.0	2913	92.3211	0.669231
RD-5285-R3-T1	RP4	105.7	2157	86.0375	1.3195
RD-5286-R3-T1	RP4	159.2	3104	85.733	1.1636
RD-5287-R3-T1	RP4	49.8	1329	56.3445	3.36284
RD-5288-R3-T1	RP4	83.3	1845	85.3378	1.20293
RD-5289-R3-T1	RP4	87.2	1756	77.1079	2.02875
RD-5290-R3-T1	RP4	155.1	2632	90.8344	0.871477
RD-5291-R3-T1	RP4	35.4	1235	88.8392	0.906933
RD-5292-R3-T1	RP4	72.0	1667	91.4023	0.749126
RD-5293-R3-T1	RP4	16.9	1221	83.7864	1.53784
RD-5294-R3-T1	RP4	33.6	1232	81.6973	1.38648
RD-5295-R3-T1	RP4	22.7	1370	87.9562	1.00781
RD-5296-R3-T1	RP4	103.4	2331	93.9305	0.543633
RD-5297-R3-T1	RP4	50.5	2818	82.2834	1.64231
RD-5298-R3-T1	RP4	23.8	1982	85.0088	1.3701

Supplementary Table 13: MSI NGS peak number and mean indel length for reproducibility runs.
See Supplementary_Table_13

Supplementary Table 14: NTC nM library yield and length across all runs

Sample ID	Run ID	Run Description	Library Yield (nM)	Library Length (Tape Station)
NTC-CV1	CV1	Validation set	2.2	149
NTC-CV2	CV2	Validation set	8.6	189
NTC-CV3	CV3	Validation set	5.7	156
NTC-CV4	CV4	Validation set	4.8	154
NTC-CV5	CV5	Validation set	7.3	147
NTC-AV1	AV1	Standard Batch size (n = 20)	2.2	149
NTC-AV2	AV2	Batch size (n = 40)	1.9	157
NTC-AV3	AV3	Batch size (n = 10)	3.8	150
NTC-AV4	AV4	Batch size (n = 5)	3.9	151
NTC-AV5	AV5	DNA input (50, 20, 10, 5 ng)	4.4	148
NTC-AV6	AV6	Percent Tumor Nuclei (70, 52.5, 35, 28, 21, 14, 7%)	1.6	218
NTC-AV7	AV7	Percent Tumor Nuclei (90, 67.5, 45, 36, 27, 18, 9%)	2.1	194
NTC-RP1	RP1	Reproducibility (Intra-Run 1)	1.8	152
NTC-RP2	RP2	Reproducibility (Inter-Run 2, tech 1)	2.2	149
NTC-RP3	RP3	Reproducibility (Inter-Run 3, tech 2)	3	148
NTC-RP4	RP4	Reproducibility (Inter-Run 4, tech 1)	8.2	150
		Mean	4	161
		Standard Deviation	2.3	22
		Mean + 2x SD	8.5	206

Supplementary Table 15: MSI-CTL and MSS-CTL indel number, mean indel length and QC parameters. See Supplementary_Table_15

Supplementary Table 16: Summary of MSI NGS run level parameters and QC thresholds

Run Name	Run Description	Parameter			
		**Density (K/mm^2)**	**Clusters PF (%)**	**Reads PF (M)**	**% ≥ Q30**
Accuracy Run 1/Analytical Validity Run 1/Reproducibility Run 2	Accuracy set	1054	88.49	10	81.51
Accuracy Run 2	Accuracy set	1111	76.91	8.82	81.86
Accuracy Run 3	Accuracy set	1096	81.75	9.46	77.39
Accuracy Run 4	Accuracy set	1064	86.52	9.8	75.45
Accuracy Run 5	Accuracy set	1086	86.17	10.02	81.21
Accuracy Run 1/Analytical Validity Run 1/Reproducibility Run 2	Standard Batch size (n = 20)	1054	88.49	10	81.51
Analytical Validity Run 2	Batch size (n = 40)	1156	83.99	10.24	81.40
Analytical Validity Run 3	Batch size (n = 10)	1100	87.07	10.28	82.70
Analytical Validity Run 4	Batch size (n = 5)	1143	85.79	10.38	83.14
Analytical Validity Run 5	DNA input (50, 20, 10, 5 ng)	1140	84.08	10.16	80.68
Analytical Validity Run 6	Percent Tumor Nuclei (70, 52.5, 35, 28, 21, 14, 7%)	1129	84.82	10.16	79.69
Analytical Validity Run 7	Percent Tumor Nuclei (90, 67.5, 45, 36, 27, 18, 9%)	1049	88.29	9.96	79.88
Reproducibility Run 1	Reproducibility (Intra-Run 1)	1069	86.45	9.72	81.08
Accuracy Run 1/Analytical Validity Run 1/Reproducibility Run 2	Reproducibility (Inter-Run 2, tech 1)	1054	88.49	10	81.51
Reproducibility Run 3	Reproducibility (Inter-Run 3, tech 2)	1117	85.22	9.98	80.40
Reproducibility Run 4	Reproducibility (Inter-Run 4, tech 1)	1022	88.75	9.76	79.48
	**AVERAGE**	1090.25	85.71	9.92	80.55
	**SD**	38.58	2.98	0.36	1.87
	**AVERAGE - 2SD**	**1013.09**	**79.74**	**9.20**	**76.82**
	**MIN**	1022	76.91	8.82	75.445
	**MAX**	1156	88.75	10.38	83.14

Supplementary Table 17: Sample level quantitation and QC data for accuracy study and performance parameter development

Sample ID	Run	Library Yield	Depth of Coverage	mapped_reads_pct	singletons_pct
RD-5289	Run1	87.1	2445	95.6963	0.5277
RD-5284	Run1	74.2	2449	96.6260	0.4643
RD-5290	Run1	88.9	2322	90.8441	0.9589
RD-5291	Run1	81.3	2656	95.9487	0.4669
RD-5292	Run1	102.3	2351	93.8072	0.7032
RD-5279	Run1	55.9	2422	95.6796	0.5621
RD-5280	Run1	24.2	1914	92.8141	0.7741
RD-5285	Run1	39.9	2033	95.1607	0.5241
RD-5281	Run1	26.5	2056	94.9346	0.6080
RD-5286	Run1	115.9	3103	95.5810	0.5386
RD-5287	Run1	18.3	1855	93.6543	0.6338
RD-5288	Run1	59.2	2497	96.9827	0.4340
RD-5282	Run1	56.7	2510	92.9368	0.7955
RD-5283	Run1	41.0	2400	96.9315	0.4015
RD-5293	Run1	37.3	2142	95.4313	0.6134
RD-5294	Run1	68.6	2443	93.2658	0.7019
RD-5295	Run1	72.9	2200	94.0390	0.6300
RD-5296	Run1	61.9	2172	94.4695	0.6064
RD-5297	Run1	72.3	2256	90.0165	1.0325
RD-5298	Run1	49.2	1883	79.9581	1.9284
RD-5302	Run2	89.2	2070	95.8442	0.4610
RD-5303	Run2	52.3	1744	89.2734	0.7584
RD-5304	Run2	67.0	1908	92.4191	0.6206
RD-5306	Run2	63.9	1759	93.3858	0.5736
RD-5307	Run2	66.9	1996	93.1930	0.5451
RD-5299	Run2	90.4	1760	94.5840	0.4801
RD-5308	Run2	47.0	1773	92.4612	0.5528
RD-5309	Run2	35.4	1958	95.0031	0.4884
RD-5300	Run2	72.8	2803	96.5043	0.3896
RD-5310	Run2	94.9	2224	94.5431	0.5075
RD-5311	Run2	54.8	1893	91.6578	0.7098
RD-5312	Run2	98.0	2196	96.4707	0.3329
RD-5313	Run2	94.1	2274	95.8438	0.3562
RD-5314	Run2	23.5	1944	93.6112	0.5666
RD-5315	Run2	44.6	1773	94.9354	0.4260
RD-5316	Run2	149.6	2608	96.8891	0.3282
RD-5317	Run2	104.9	3485	97.3530	0.2983
RD-5318	Run2	96.1	2844	94.2347	0.5668
RD-5319	Run2	89.0	2824	95.6868	0.4439
RD-5301	Run2	29.0	1510	91.1965	0.7982
RD-5320	Run3	56.9	2080	89.4068	0.8199
RD-5321	Run3	29.6	3061	94.8674	0.4205
RD-5322	Run3	25.7	1599	83.9720	1.2015
RD-5323	Run3	98.4	2574	97.5283	0.2924
RD-5324	Run3	78.7	2275	92.7665	0.5371
RD-5325	Run3	41.1	1925	92.6724	0.6175
RD-5326	Run3	48.7	2496	95.3086	0.4872
RD-5327	Run3	24.8	1467	89.9646	0.7283
RD-5328	Run3	27.4	1405	88.5308	0.8478
RD-5329	Run3	23.7	2576	94.1691	0.4766
RD-5330	Run3	57.1	2488	94.1054	0.5153

RD-5331	Run3	20.3	1618	83.7121	1.3055
RD-5332	Run3	58.2	1603	79.8898	1.4677
RD-5333	Run3	27.9	731	39.7917	4.3887
RD-5334	Run3	6.8	770	80.9950	1.6618
RD-5335	Run3	18.7	936	78.1309	1.5849
RD-5336	Run3	49.5	1994	95.7611	0.3776
RD-5337	Run3	23.1	1722	88.5739	0.8375
RD-5338	Run3	52.1	1554	72.4677	2.1349
RD-5339	Run3	54.6	1999	90.1253	0.8890
RD-5344	Run4	120.7	2701	90.8337	0.6765
RD-5345	Run4	55.2	1864	85.3128	1.0213
RD-5340	Run4	39.8	1883	84.1275	1.0109
RD-5341	Run4	21.7	2294	90.7748	0.6156
RD-5342	Run4	24.7	1817	89.9862	0.7216
RD-5346	Run4	64.6	2499	89.6596	0.8407
RD-5350	Run4	12.4	1227	72.2236	1.7579
RD-5351	Run4	51.6	1683	72.5801	1.8459
RD-5343	Run4	10.1	916	76.1318	1.5568
RD-5347	Run4	37.6	925	67.0085	1.9294
RD-5352	Run4	14.8	738	87.4512	0.7545
RD-5353	Run4	45.1	1304	90.6511	0.6064
RD-5354	Run4	7.2	1135	74.3379	1.8835
RD-5355	Run4	28.4	1841	91.8235	0.5887
RD-5356	Run4	5.4	1269	83.1393	1.0796
RD-5357	Run4	24.3	1423	90.6523	0.6720
RD-5358	Run4	18.7	915	82.4122	1.0721
RD-5359	Run4	7.5	1037	72.9838	1.7214
RD-5348	Run4	18.6	735	51.2817	2.8618
RD-5349	Run4	19.6	1055	70.1749	1.8667
RD-5370	Run5	95.0	2760	95.8500	0.3509
RD-5379	Run5	9.8	1736	70.7076	2.5731
RD-5380	Run5	40.1	2263	86.0271	1.2503
RD-5369	Run5	47.6	1978	91.4740	0.6302
RD-5371	Run5	31.4	1991	91.6757	0.7474
RD-5372	Run5	11.6	1863	84.3992	1.3962
RD-5373	Run5	75.0	2393	94.5042	0.4462
RD-5374	Run5	94.5	2675	93.2288	0.5616
RD-5375	Run5	27.3	2058	93.2955	0.6381
RD-5376	Run5	15.2	2050	81.5171	1.4221
RD-5377	Run5	68.2	2921	92.5768	0.6135
RD-5378	Run5	103.7	3141	95.1563	0.4346
RD-5360	Run5	77.5	1968	91.1661	0.7040
RD-5361	Run5	101.0	2158	92.8090	0.5722
RD-5363	Run5	99.7	2971	92.3334	0.7342
RD-5362	Run5	40.4	2223	89.6913	0.8471
RD-5364	Run5	71.2	2225	93.5225	0.5050
RD-5365	Run5	66.4	1959	92.4599	0.6009
RD-5366	Run5	53.6	1810	92.9167	0.6605
RD-5368	Run5	74.0	2449	92.1690	0.6231
	Average	54	2012	89.0363	0.8710
	Standard Deviation	31	582	9.4420	0.6194
	MIN	5	731	39.7917	0.2924
	MAX	150	3485	97.5283	4.3887
	Average +/- 2(3) SD		**> 500**	**70.1524**	**1.7481**

Supplementary Table 18: List of samples in the gold standard 100 cohort with tumor type, site of tissue collection, tumor characteristics and PCR/IHC data. See Supplementary_Table_18

Supplementary Table 19: MSI NGS vs. MSI-PCR concordance for accuracy study. See Supplementary_Table_19

Supplementary Table 20: Summary of MSI-NGS quality control parameters

Test Mode	Level	Parameter	Application	Rule	Metric	Value
MSI-NGS	Sample	Standard ng input	Threshold	≥ 20 ng yield	ng	≥ 20
MSI-NGS	Sample	mapped_reads_pct	Threshold	≥ mean -2SD from validation	Percentage	≥ 70.5
MSI-NGS	Sample	singletons_pct	Threshold	≥ mean +3SD from validation	Percentage	≤ 1.75
MSI-NGS	Sample	Coverage	Threshold	≥ 500x per validation	Number	≥ 500
MSI-NGS	Sample	Sample library generation yield	Trend/Threshold	≥ NTC mean +2SD from validation	nM	≥ 8.5
MSI-NGS	Run	NTC DNA Run Control library Construct	Trend/Threshold	Amplicon size	bp	< 300
MSI-NGS	Run	MSI-CTL Prediction score/ Cluster assignment	Trend/Threshold	2	Score	MSI
MSI-NGS	Run	MSS-CTL Prediction score/ Cluster assignment	Trend/Threshold	3	Score	MSS
MSI-NGS	Run	Run cluster density	Trend/Threshold	Predefined Illumina metric	Density (K/mm2)	500K–1300K
MSI-NGS	Run	Run clusters passing filter	Trend/Threshold	≥ mean -2SD from validation	Clusters PF (%)	≥ 79.74
MSI-NGS	Run	Total reads passing filter	Trend/Threshold	≥mean -2SD from validation	Reads PF (M)	≥ 9.20
MSI-NGS	Run	Probability of Incorrect Base Call	Trend/Threshold	≥ mean -2SD from validation	% ≥ Q30 Average (Read 1, Read 4)	≥ 76.82
